# Blood Poisoning after Tooth Extraction

**Published:** 1897-03

**Authors:** 


### Blood Poisoning after Tooth Extraction.

Two cases have lately come under our notice of fatal blood-poisoning occurring after tooth-extraction. In one case, that of a sailor in New South Wales, it seems that the patient had applied some lotion to the afflicted part. Medical witnesses differed as to whether this lotion would have a deleterious effect; one medical man saying that it was harmless, while another affirmed that if the man had not used it, he would still have been alive. In the other case, that of a boy whose tooth was extracted by a chemist, at Framlingham, it appears that the lad had lanced his own gum with a pocket-knife, and a verdict of death from natural causes was returned. We can not be too careful in seeing that all our instrument, especially forceps, are thoroughly well cleaned after each operation, as well for our own protection as for that of the public.—*Brit. Jour. Dental Science.*

